# MEK1/2 inhibition decreases pro-inflammatory responses in macrophages from people with cystic fibrosis and mitigates severity of illness in experimental murine methicillin-resistant *Staphylococcus aureus* infection

**DOI:** 10.3389/fcimb.2024.1275940

**Published:** 2024-01-30

**Authors:** Mithu De, Gregory Serpa, Eryn Zuiker, Katherine B. Hisert, W. Conrad Liles, Anne M. Manicone, Emily A. Hemann, Matthew E. Long

**Affiliations:** ^1^ Department of Internal Medicine, Division of Pulmonary, Critical Care, and Sleep Medicine, The Ohio State University, Columbus, OH, United States; ^2^ Department of Microbial Infection and Immunity, The Ohio State University, Columbus, OH, United States; ^3^ Department of Medicine, National Jewish Health, Denver, CO, United States; ^4^ Department of Medicine, Division of Infectious Diseases, University of Washington, Seattle, WA, United States; ^5^ Center for Lung Biology, University of Washington, Seattle, WA, United States; ^6^ Department of Medicine, Division of Pulmonary, Critical Care, and Sleep Medicine, University of Washington, Seattle, WA, United States

**Keywords:** cystic fibrosis, macrophage, neutrophil, *S. aureus*, therapeutic

## Abstract

Chronic pulmonary bacterial infections and associated inflammation remain a cause of morbidity and mortality in people with cystic fibrosis (PwCF) despite new modulator therapies. Therapies targeting host factors that dampen detrimental inflammation without suppressing immune responses critical for controlling infections remain limited, while the development of lung infections caused by antimicrobial resistant bacteria is an increasing global problem, and a significant challenge in CF. Pharmacological compounds targeting the mammalian MAPK proteins MEK1 and MEK2, referred to as MEK1/2 inhibitor compounds, have potential combined anti-microbial and anti-inflammatory effects. Here we examined the immunomodulatory properties of MEK1/2 inhibitor compounds PD0325901, trametinib, and CI-1040 on CF innate immune cells. Human CF macrophage and neutrophil phagocytic functions were assessed by quantifying phagocytosis of serum opsonized pHrodo red *E. coli*, *Staphylococcus aureus*, and zymosan bioparticles. MEK1/2 inhibitor compounds reduced CF macrophage pro-inflammatory cytokine production without impairing CF macrophage or neutrophil phagocytic abilities. Wild-type C57BL6/J and *Cftr* ^tm1kth^ (F508del homozygous) mice were used to evaluate the *in vivo* therapeutic potential of PD0325901 compared to vehicle treatment in an intranasal methicillin-resistant *Staphylococcus aureus* (MRSA) infection with the community-acquired MRSA strain USA300. In both wild-type and CF mice, PD0325901 reduced inflammation associated body mass loss. Wild-type mice treated with PD0325901 had significant reduction in neutrophil-mediated inflammation compared to vehicle treatment groups, with preserved clearance of bacteria in lung, liver, or spleen 1 day after infection in either wild-type or CF mouse models. In summary, this study provides the first data evaluating the therapeutic potential of MEK1/2 inhibitor to modulate CF immune cells and demonstrates that MEK1/2 inhibitors diminish pro-inflammatory responses without impairing host defense mechanisms required for acute pathogen clearance.

## Introduction

Mutations in the cystic fibrosis transmembrane conductance regulator (CFTR) that impair this ion channel’s function result in the disease cystic fibrosis (CF). CF has many pathophysiological manifestations; however, CF pulmonary disease remains the main driver of morbidity and mortality. Decades of investment in preclinical research have recently culminated in breakthrough new therapeutic highly effective modulator therapies (HEMT) that restore CFTR function and have rapidly changed the landscape of CF clinical disease and outcomes. However, despite the significant improvements in a majority of people with CF (PwCF) who have access to HEMT, established chronic bacterial colonization is not eradicated in many PwCF receiving modulator therapy, and ongoing airway infection is anticipated to remain a healthcare challenge for PwCF (Hisert et al., 2017; Sheikh et al., 2023). In addition, there remains a significant minority of PwCF who are not eligible for or do not tolerate current modulator therapies in whom improved therapies to mitigate lung damage caused by chronic airway infection and inflammation are still needed. Novel strategies to alleviate pulmonary inflammation and infection are essential to address these gaps in care for PwCF.

Innate immune cells, including monocytes, macrophage, and neutrophils, that reside in the lung or are recruited during infection or injury are critical for eliminating infections and resolving the inflammatory response. However, the accumulation of inflammatory cells in the CF lung, especially neutrophils, contributes to tissue pathology (Rosen et al., 2018), and therapeutic approaches to reduce the tissue damaging effects of innate immune cells in the CF lung have been elusive. A phase 2 clinical trial of a prior anti-inflammatory therapy developed to blunt neutrophil recruitment (a leukotriene B4 inhibitor) and thus damage to the CF airway was halted early due to participants experiencing increased pulmonary exacerbations (Konstan et al., 2014). Subsequent murine studies demonstrated that decreased neutrophils in the lung allowed for increased growth of *P. aeruginosa* (Doring et al., 2014). These unexpected outcomes highlight the importance of pre-clinical testing of anti-inflammatory therapies to ensure that suppression of specific arms of the inflammatory response do not inadvertently dampen anti-microbial responses. Our previous studies and others’ have demonstrated that targeted inhibition of mammalian protein kinases MEK1/2 is a promising therapeutic strategy to reduce detrimental inflammation during the context of lung injury and infection (Schuh and Pahl, 2009; Long et al., 2017a; Long et al., 2017b). MEK1 and MEK2 are serine/threonine protein kinases that heterodimerize upon activation and then phosphorylate ERK1/2, among others, as a main downstream target in cellular signaling pathways. The MEK1/2-ERK1/2 pathway is a central signaling hub for multiple cell surface receptors, including TLRs, and the development of small molecules to inhibit the MEK1/2-ERK1/2 pathway has taken place over the last several decades predominately due to the high levels of over-activation of this pathway in several types of human cancer (Zhao and Adjei, 2014). Four different MEK1/2 inhibitor compounds are currently FDA approved, with several additional compounds under investigation in clinical trials (Roskoski, 2022). Furthermore, novel therapeutic applications of MEK1/2 inhibitor compounds, including administration during respiratory viral infections, is an active area of preclinical and translational research (Droebner et al., 2011; Schreiber et al., 2022a; Schreiber et al., 2022b). The current study was undertaken to determine whether MEK1/2 inhibitor compounds can reduce phagocyte-mediated inflammation in CF without impairing phagocyte host defense mechanisms. *Pseudomonas aeruginosa* and *Staphylococcus aureus* are the two most common airway colonizers in CF. Therefore, this study utilized primary human blood monocyte-derived macrophages and neutrophils from PwCF to examine how administration of MEK1/2 inhibitor compounds modulate CF macrophage TLR4 or TLR2-dependent pro-inflammatory responses and CF macrophage and neutrophil phagocytic capacity. Wild-type C57BL6/J and *Cftr* ^tm1kth^ (F508del homozygous) mice were used to evaluate if therapeutic dampening of the *in vivo* inflammatory response by a MEK1/2 inhibitor impaired host defense following acute pulmonary infection with methicillin-resistant *Staphylococcus aureus* (MRSA).

## Methods

### Reagents

The MEK1/2 inhibitor compounds PD0325901 (catalog # S1036), CI-1040 (catalog # S1020), and trametinib (catalog # S2673) were purchased from Selleck for use in *in vitro* cell culture experiments. PD0325901 and trametinib were used at 0.5 μM and CI-1040 was used at 10 μM. PD0325901 (catalog # PZ0162) used for *in vivo* experiments was from Sigma. Recombinant human M-CSF (catalog # 300-25) and murine M-CSF (catalog # 315-02) were from Peprotech. Ficoll Paque Plus (catalog # 17-1440-03) was from Cytiva. Human AB serum was from Sigma (catalog # H6914). LPS from *Pseudomonas aeruginosa* was from Sigma (catalog # L8643). Pam3CSK4 (catalog # tlrl-pms) and FSL1 (catalog # tlrl-fsl) were from InvivoGen (San Diego, CA). The pHrodo Red *E. coli* (catalog # P35361), *S. aureus* (catalog # A10010), and zymosan (catalog # P35364) bioparticles were from ThermoFisher Scientific. Antibodies used in these studies are listed in **Supplementary Table 1**.

### Animal ethics and mouse infection

The Institutional Animal Care and Use Committee at The Ohio State University approved all studies (OSU IACUC #2020A00000081). *Cftr* ^tm1kth^ F508del homozygous mice were acquired from the Case Western University Cystic Fibrosis Mouse Model Core. Mice were maintained ad libitum on Golytely drinking water (25 mM sodium chloride, 10 mM potassium chloride, 40 mM sodium sulfate, 20 mM sodium bicarbonate, 18 mM PEG 3350). C57BL/6J wild-type mice were obtained from Jackson Laboratories. For infection, an overnight culture of methicillin-resistant *S. aureus* USA300 grown in BHI was sub-cultured to mid-logarithmic phase. Bacteria were washed twice with sterile PBS and diluted so that 1 x 10^7^ colony forming units (CFU) were delivered in a 50 μl inoculum. Mice were anesthetized by i.p. injection of ketamine/xylazine. Anesthetized mice were provided i.p. injection of sterile PBS containing the MEK1/2 inhibitor PD0325901 (20 mg/kg) or vehicle (DMSO) immediately (within 5 minutes) prior to intranasal infection. Infection in the anesthetized mice was performed by intranasal delivery of a 50 μl inoculum. Animals were monitored for recovery from anesthesia and weight loss was measured daily. Groups of mice were sacrificed 24 hours after infection and subjected to broncho-alveolar lavage (BAL) using sterile PBS containing 2 mM EDTA. BAL cells were isolated by centrifugation at 350 x *g* for 10 minutes at 4°C. BAL fluid (BALF) was stored at -80°C until use in ELISA assays. Total cell counts were performed on BAL cells and the total neutrophils and macrophage/monocytes were quantified from differential staining of cytospin preparations (Long et al., 2019). Lung, liver, and spleens were collected, homogenized in 1 mL of sterile PBS with the Precellys lysing kit beads (catalog # P000912-LYSK1-A), and serial dilutions of tissue homogenates were plated on TSA and incubated overnight at 37°C to allow colony growth. Colonies were enumerated and the total CFU recovered were calculated and normalized per gram of tissue collected. For histology, a lung section was immersed in 10% formalin for 24 hours and changed to 30% ethanol then 50% ethanol before final storage 70% ethanol; processing and IHC was performed by Histowiz. For quantification of the percentage of total lung area CD45-positive, ten random images at 10x magnification were selected and quantification was performed on ImageJ.

### Cell culture

Human subjects research was approved by the Institutional Review Board at Nationwide Children’s Hospital and The Ohio State University. Blood donors were PwCF consented at Nationwide Children’s Hospital by clinical research coordinators supported by the Cure CF Columbus Research Development Program. Human blood was collected into EDTA-vacutainer tubes following approved IRB protocols and samples were de-identified. Demographic information of blood donors is listed in **Supplementary Table 1**. Peripheral blood mononuclear cells (PBMC) were isolated by Ficoll gradient centrifugation and differentiated to monocyte-derived macrophages for 7-10 days by incubation with 20 ng/mL recombinant human M-CSF. Macrophages were seeded into 24-well tissue culture plates at a density of 300,000 cells/well in HEPES-buffered RPMI-1640 containing 10% HI-FBS, penicillin/streptomycin, L-glutamine. Neutrophils were also isolated from peripheral blood by Ficoll gradient centrifugation and used immediately following isolation (Nauseef, 2014). Bone-marrow derived macrophages (BMDM) were isolated and cultured as previously described (Long et al., 2017a). Briefly, bone marrow was collected from the femur and tibia of wild-type mice and differentiated to macrophages over 7 days by culture in media containing 20 ng/mL recombinant murine M-CSF. BMDM were collected from petri dishes and plated at 300,000 cells per well in 24-well plates and allowed to adhere overnight before initiation of treatment or stimulations.

### Phagocytosis of pHrodo bioparticles

For opsonization of pHrodo labeled bioparticles, a frozen vial was resuspended in sterile HBSS containing calcium and magnesium (according to manufacturer’s recommendations) and placed in a water bath sonicator for 15 minutes to fully disperse particles. To opsonize the bioparticles, an equal volume of human serum was added to the particles and were incubated for 30 minutes at 37°C. Following opsonization, bioparticles were added to human macrophage or neutrophil cultures at a concentration of 0.08 mg/mL in media containing either a vehicle (DMSO), PD0325901, Trametinib, CI-1040, or Cytochalasin D. Neutrophil cultures were gently tumbled for 3 minutes at 37°C to ensure proper mixing followed by subsequent static incubation. Following a 1 hour incubation at 37°C, supernatants were aspirated and cells were washed twice with PBS to remove non-ingested particles. Macrophages were collected by addition of PBS containing 2 mM EDTA to the cultures and cells were gently lifted using a cell scraper, washed with cell staining buffer (catalog # 420201, BioLegend), and stored on ice and protected from light (Gharib et al., 2019). Data were collected on a Becton Dickinson LSRFortessa flow cytometer and analyses performed with FlowJo software. Cytochalasin D was added to samples at 2 μM as a control for inhibition of phagocytosis and was used to draw flow gates to identify the percent of cells negative and positive for pHrodo red. In separate experiments, murine BMDM were used to evaluate whether pre-treatment of BMDM for 24 hours with either vehicle, PD0325901, Trametinib, or CI-1040 altered phagocytosis of bioparticles compared to BMDM that were treated with these compounds only during the 1 hour phagocytosis incubation period.

### Western blot and ELISA

*In vitro* stimulations of human monocyte-derived macrophages from PwCF were performed for 4 hours and the protein lysates were prepared as previously described (Long et al., 2017a). In brief, RIPA lysis buffer containing protease and phosphatase inhibitors (Life Technologies) were added to cells on ice for 20 minutes. Cell lysates were scraped from the wells, collected into pre-chilled centrifuge tubes and the lysates were cleared by centrifugation at 14,000 x *g* for 5 minutes at 4°C. Quantitation of protein lysates were performed using a BCA assay (Life Technologies), and samples were prepared to load 5-10 ug of total protein per lane of a gel. Antibodies used for western blots are listed in **Supplementary Table 2**. Supernatants from stimulated cells were collected and centrifuged at 14,000 x *g* for 5 minutes to clear cellular debris and were stored at -80°C prior to ELISA analysis. Human IL-8/CXCL8, TNF, IL10, IL1β, and IL-12 DuoSet ELISA (R&D Systems) were used to quantify levels of cytokines and chemokines in cell culture supernatants. All ELISA kits are listed in **Supplementary Table 3**. LPS stimulations were performed at 50 ng/mL, Pam3CSK4 at 1 μg/mL and FSL1 at 100 ng/mL.

### Quantitative RT-PCR

RNA was isolated from murine BMDM 4 hours after stimulations using the NucleoSpin RNA isolation kit (catalog # 740995.250, Takara Bio USA, Inc). RNA concentrations were quantified using a NanoDrop 1000 (Thermo Fisher Scientific), and equal amounts of RNA from each sample were used to synthesize cDNA using the High-Capacity cDNA Reverse Transcription Kit (catalog # 4368813, Applied Biosystems). TaqMan Fast Advanced Master Mix (catalog # 4444557, Applied Biosystems) and primer probes (**Supplementary Table 4**) were used with cDNA templates for quantitative real-time PCR (qPCR). Reactions and data collection were performed on an Applied Biosystems StepOnePlus Real-time PCR System using Software V2.2 and the Ct was used to quantify the fold change in relative expression compared to unstimulated control samples from an individual experiments using 2^-∆∆Ct^ calculations (Long et al., 2017a).

## Results

### Inhibition of the MEK1/2 pathway decreases CF macrophage TLR-induced IL-1β production.

**Figure 1:**
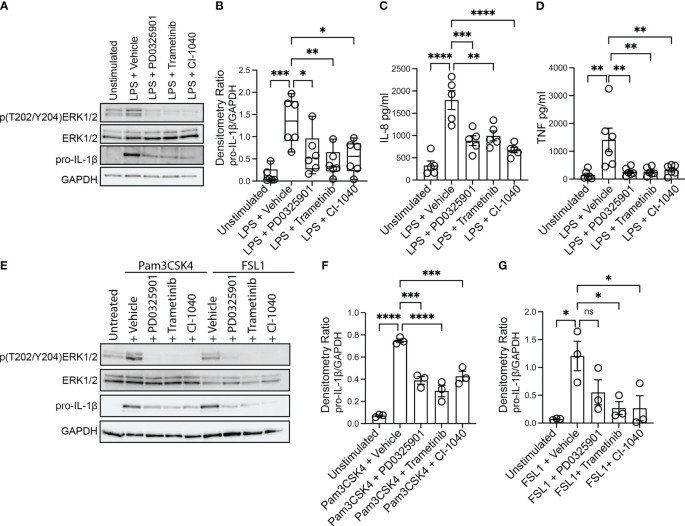
MEK1/2 inhibitors reduce CF macrophage TLR4 and TLR2 pro-inflammatory responses. Human CF macrophages were stimulated with 50 ng/mL of *P. aeruginosa* LPS **(A-D)**, 1 μg/mL Pam3CSK4 **(E, F)** or 100 ng/mL FSL1 **(E, G)** for 4 hours with the addition of vehicle, PD0325901, Trametinib, or CI-1040 to media at the initiation of stimulation. **(A)** Protein lysates from a representative experiment from one donor, **(B)** densitometry quantitation of the ratio of pro-IL-1 to GAPDH from n=6 donors. **(C)** Measurement of the levels of IL-8 or **(D)** TNF from supernatants collected at 4 hours from n=5-6 donors. **(E)** Protein lysates from one representative experiment of three; **(F, G)** densitometry quantitation from n=3 donors. **(B, C, D, F, G)** Data are the mean ± SEM, each point represents one individual donor. Statistical analyses were performed by One-Way ANOVA with Tukey multiple comparisons, * *P<*0.05, ** *P<*0.01, *** *P<*0.001, **** *P<*0.0001, ns is not significant.

Production and secretion of IL-1β and IL-8 are thought to be key drivers of pulmonary inflammation in CF (Roesch et al., 2018). Bacterial infections persist in the CF airway despite a robust and exuberant immune response. The majority of immune cells that are present in the CF airways are phagocytes (monocyte-derived macrophages and neutrophils) recruited from blood (Schupp et al., 2020), and recent work indicates that inflammatory damage caused by the recruited cells is dependent on monocyte derived macrophages (Oz et al., 2022). We thus sought to evaluate whether inhibition of the MEK1/2 pathway could decrease deleterious inflammatory responses generated by CF monocyte-derived macrophages. To test the hypothesis that MEK1/2 inhibitor compounds would reduce production of pro-IL-1β and secretion of IL-8, CF macrophages were stimulated with *P. aeruginosa* LPS in media containing vehicle or one of the MEK1/2 inhibitor compounds, PD0325901, Trametinib, or CI-1040. When analyzed 4 hours after stimulation, addition of the MEK1/2 inhibitor compounds reduced MEK1/2-ERK1/2 pathway activation, as measured by reduced ERK1/2(pT202/pT204) (**Figure 1A**), and the LPS-induced production of pro-IL-1β protein was also significantly reduced by addition of a MEK1/2 inhibitor compound compared to vehicle-treated control (**Figures 1A, B**). Further, the LPS-induced secretion of IL-8 (**Figure 1C**) and TNF (**Figure 1D**) were significantly reduced by addition of the MEK1/2 inhibitor compounds compared to vehicle-treated samples. The overall secretion of IL-1β was low, consistent with priming and activation steps necessary for inflammasome-dependent activation of caspase-1 and cleavage of pro-IL-1β. However, the addition of MEK1/2 inhibitors resulted in a trend towards a reduction in secreted IL-1β, **(Supplementary Figure 1A**), consistent with a mechanism of reduced transcription of *Il1b* (**Supplementary Figure 1E**). As PwCF have frequent *S. aureus* infections in addition to *P. aeruginosa*, the ability of MEK1/2 inhibitors to reduce TLR2/1 and TLR2/6 dependent production of pro-IL-1β was assessed as these are the primary TLR receptors stimulated by gram-positive bacteria. Similar to results following LPS stimulation, addition of MEK1/2 inhibitor compounds reduced pro-IL-1β protein levels following Pam3CSK4 (ligand of TLR2/1) (**Figure 1E, F**) or FSL1 (ligand of TLR2/6) (**Figures 1E, G**) stimulations.

While the MEK1/2 inhibitor mediated reduction in TLR4 and TLR2 pro-inflammatory cytokines and chemokines may have anti-inflammatory effects, decreased amounts of other cytokines, such as IL-10 or IL-12, may alter systemic inflammation or protective immune responses to *S. aureus* infections (Nguyen et al., 2015; Leech et al., 2017). Given this, we measured secretion of IL-10 and IL-12 from CF macrophage cultures. While MEK1/2 inhibitors had a moderate but not statistically significant reduction in LPS-induced IL-10 secretion (**Supplementary Figure 1B**), IL-10 was significantly reduced by MEK1/2 inhibitors following TLR2/1 stimulation with Pam3CSK4 (**Supplementary Figure 1C**), and moderately reduced by MEK1/2 inhibitors following TLR2/6 stimulation (**Supplementary Figure 1D**). In contrast, the levels of IL-12p70 secreted were below the limit of detection in all samples (data not shown). To further examine whether IL-12 was altered by MEK1/2 inhibitors, we utilized murine BMDM as a model to quantify whether MEK1/2 inhibitors altered expression of *Il12a* or *Il12b* following either LPS, Pam3CSK4, or FSL1 stimulation. Induction of *Il1b and Il10* expression by LPS was decreased with addition of MEK1/2 inhibitor compounds (**Supplementary Figures 1E-F**). However, expression of *Il12a* or *Il12b* was not altered by MEK1/2 inhibitors added during LPS (**Supplementary Figures 1G-H**), Pam3CSK4 (**Supplementary Figures 1I-J**) or FSL1 (**Supplementary Figures 1K-L**) stimulation. Together, these results demonstrate that MEK1/2 inhibitors significantly reduce CF macrophage production of a subset of both TLR4- and TLR2-dependent pro-inflammatory cytokines associated with neutrophil recruitment and tissue damage.

### MEK1/2 inhibitors do not impair CF macrophage or neutrophil phagocytosis

**Figure 2:**
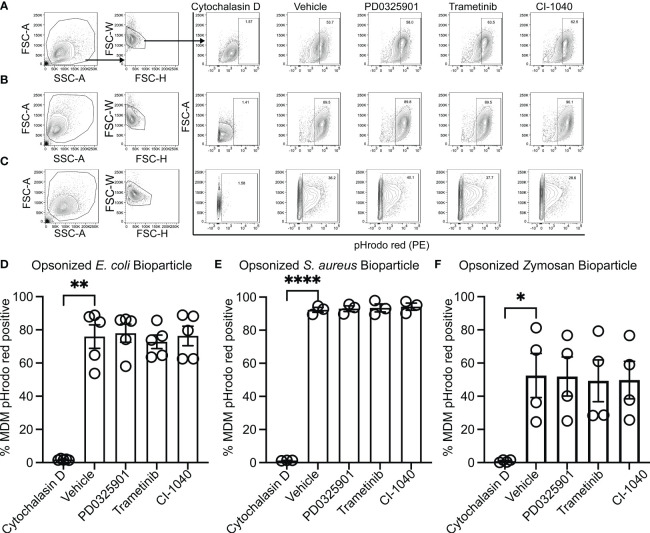
MEK1/2 inhibitors do not impair CF macrophage phagocytosis. Representative flow cytometry gating used for phagocytosis of **(A)** pHrodo Red *E coli* bioparticles, **(B)** pHrodo Red *S. aureus* bioparticles, or **(C)** pHrodo Red Zymosan bioparticles. Quantitation of the percent of macrophages positive for pHrodo red fluorescence following incubation with serum opsonized **(D)** pHrodo Red *E coli* bioparticles, **(E)** pHrodo Red *S. aureus* bioparticles, or **(F)** pHrodo Red Zymosan bioparticles. Cells were exposed to vehicle, MEK1/2 inhibitor compounds, or cytochalasin D during the 1 hour phagocytosis incubation period with pHrodo red bioparticles. Data are the mean ± SEM and each point represents data from one individual donor. Statistical analyses were performed by One-Way ANOVA with Tukey multiple comparisons, * *P<*0.05, ** *P<*0.01, **** *P<*0.0001.

**Figure 3:**
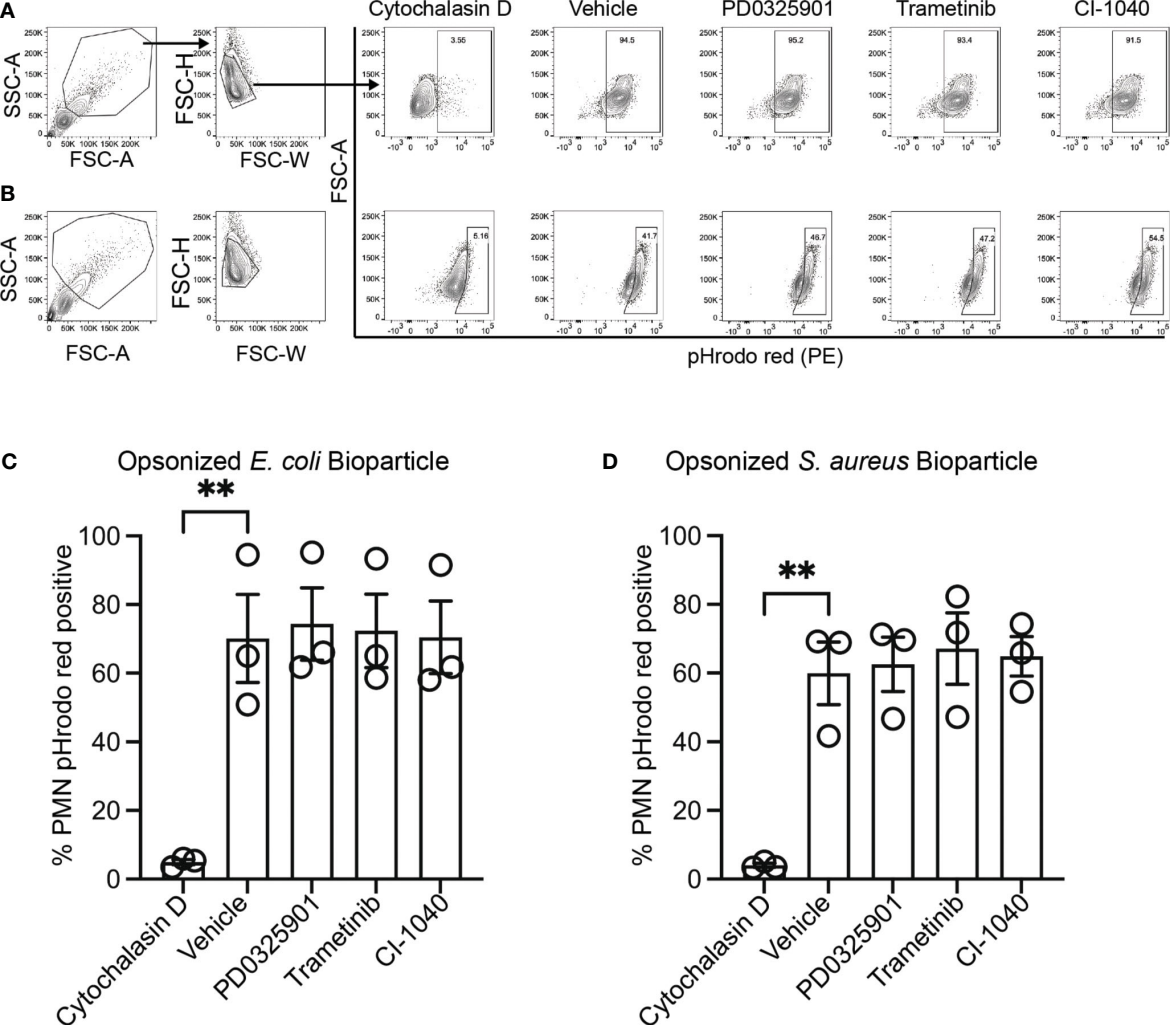
MEK1/2 inhibitors do not impair CF neutrophil phagocytosis. Representative flow cytometry gating used for phagocytosis of **(A)** opsonized pHrodo red *E. coli* bioparticles or **(B)** opsonized pHrodo red *S. aureus* bioparticles. Quantitation of the percent of neutrophils positive for pHrodo red fluorescence following incubation with opsonized **(C)** opsonized pHrodo red *E. coli* bioparticles or **(D)** opsonized pHrodo *S. aureus* bioparticles. Cells were exposed to vehicle, MEK1/2 inhibitor compounds, or cytochalasin D during the 1 hour phagocytosis incubation period with pHrodo red bioparticles. Data are the mean ± SEM and each point represents one individual donor. Statistical analyses were performed by One-Way ANOVA with Tukey multiple comparisons, ** *P*<0.01.

While production and secretion of cytokine and chemokines by macrophages modulate the inflammatory response, phagocytosis and phagosome acidification are critical cellular functions of both macrophage and neutrophils involved in host defense and homeostasis. To determine if MEK1/2 inhibitors reduced the ability of macrophage to perform these functions on gram-negative, gram-positive, or fungal microbes, phagocytosis and phagosome acidification were assayed with serum-opsonized pHrodo-labeled *E. coli* (**Figures 2A, D**), *S. aureus* (**Figures 2B, E**) or zymosan (**Figures 2C, F**) bioparticles. The pHrodo label requires acidification of the phagosome compartment to increase fluorescence, therefore detection in this assay is dependent on both bioparticle internalization and phagosome maturation. The addition of cytochalasin-D during the phagocytosis assay was used as a control and significantly reduced and prevented bioparticle ingestion, in contrast to vehicle-treated macrophage, which exhibited robust phagocytic abilities as measured by the percent of macrophages positive for pHrodo-red labeling (**Figures 2A-F**). Macrophages treated with PD0325901, Trametinib, or CI-1040 did not significantly reduce phagocytosis of either opsonized *E. coli* or opsonized *S. aureus* bioparticles compared to vehicle-treated controls. In addition, the ability of CF neutrophils to phagocytose serum-opsonized *E. coli* (**Figures 3A, C**) or *S. aureus* (**Figures 3B, D**) pHrodo bioparticles was not impacted by addition of MEK1/2 inhibitors. To evaluate whether a prolonged incubation with MEK1/2 inhibitors could result in different effects on phagocytosis due to cell surface receptor changes, wild-type BMDM were incubated with MEK1/2 inhibitors for 24 hours prior to conducting phagocytosis assays. BMDM pre-treated with MEK1/2 inhibitors for 24 hours prior to and including the 1 hour incubation with bioparticles did not have significant differences in the ability to phagocytose opsonized *E. coli* (**Supplementary Figure 2A**), opsonized *S. aureus* (**Supplementary Figure 2B**), or opsonized zymosan (**Supplementary Figure 2C**) compared to vehicle-treated samples or compared to BMDM that only received treatment during the 1-hour phagocytosis incubation period. Combined, these data are consistent with results using human CF macrophages and indicate that inhibition of MEK1/2 does not impact human or mouse macrophage or human neutrophil phagocytosis of opsonized microbes, phagosome maturation, or phagosome acidification.

### *In vivo* MEK1/2 inhibitor delivery does not impair host defense during acute MRSA pulmonary infection

**Figure 4:**
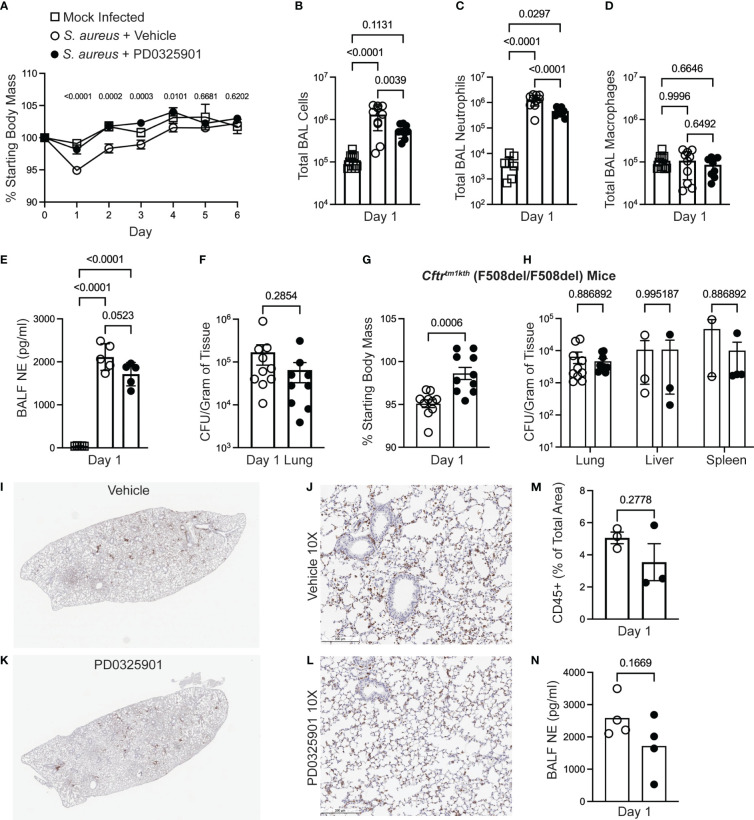
MEK1/2 inhibitor administration does not impair host defense of mice during *S. aureus* infection. Anesthetized mice were provided i.p. treatment with 20 mg/kg PD0325901 (black filled circles) or vehicle control (open circles) immediately prior to intranasal inoculation with 1 x 10^7^ CFU of *S. aureus* USA300; mock-infected (open square) animals received sterile PBS by intranasal instillation. **(A)** Body mass of C57BL6/J mice was measured following infection or mock infection for six days. Data are the mean SEM from n=5-10 males and n=5-10 females for each infected treatment group, and n=2-7 males and n=2-7 females for mock infected groups across the six day period. On day 1 after infection, **(B)** total BAL cells, **(C)** total BAL neutrophils, and **(D)** total BAL macrophages were quantified, **(E)** neutrophil elastase (NE) in BAL fluid (BALF) was quantified by ELISA, and **(F)** CFU burdens in lung homogenates were enumerated and normalized to gram of tissue collected; data points represent an individual mouse, n=9-10 mice per group combined from two independent experiments. In separate experiments **(G)** body mass of *Cftr* ^tm1kth^ F508del homozygous mice was measured on day 1 after infection, and **(H)** CFU burdens in lung, liver, and spleen homogenates were enumerated. Data points represent an individual mouse and are combined from three independent experiments; data points were omitted from the graphs if the CFU recovery was below the limit of detection (99 CFU/mL) prior to normalization to gram of tissue. **(I-L)** IHC staining for CD45 on representative lungs from a **(I-J)** vehicle-treated or **(K-L)** PD0325901-treated CF mice. **(M)** Percent of total lung area positive for CD45 staining quantified by ImageJ Software. **(N)** NE in BALF of CF mice 1 day after infection quantified, n=4 per group. Statistical analyses were performed with **(A)** Two-Way ANOVA with multiple comparisons with results shown comparing *S. aureus* + vehicle to *S. aureus* + PD0325901 groups, **(B-E)** One-Way ANOVA with Tukey’s multiple comparisons, **(F, G, M, N)** Unpaired t-test, and **(H)** multiple unpaired t-tests.

Inhibition of innate immune inflammatory functions may decrease over-exuberant inflammation seen in CF (Bruscia et al., 2009; Fan et al., 2022; Oz et al., 2022), but could result in impaired host defense response during *in vivo* infections (Doring et al., 2014; Konstan et al., 2014). Although our finding that MEK1/2 inhibition does not impair phagocytosis of microbial particles or phagosome maturation, events at a cellular level may not predict how dampening specific inflammatory pathways may affect pathogen clearance in the host. We thus sought to interrogate whether the anti-inflammatory effects of a MEK1/2 inhibitor compound would increase bacterial replication or dissemination *in vivo*. Wild-type mice were treated i.p. with either vehicle or PD0325901 immediately prior to intranasal infection with the MRSA strain USA300. At 24 hours after infection, wild-type mice receiving PD0325901-treatment had significantly less body mass loss, used as an indicator of systemic inflammation and illness, compared to vehicle-treated animals, which was sustained through day 4 of infection (**Figure 4A**). By days 5-6 after infection, all mice had returned to similar levels of body mass as mock control. Groups of mice were subjected to BAL on day 1, revealing that compared to mock-infected, vehicle-treated *S. aureus* infected mice had a significant increase in total BAL cells (**Figure 4B**), total BAL neutrophils (**Figure 4C**), without changes in total BAL macrophages (**Figure 4D**). Consistent with an anti-inflammatory effect, PD0325901-treatment of *S. aureus* infected mice significantly reduced both the total number BAL cells (**Figure 4B**) and total BAL neutrophils (**Figure 4C**) compared to vehicle-treated *S. aureus* infected mice without alteration of total BAL macrophage levels. Infected mice had a significant elevation in neutrophil elastase in the BAL fluid (BALF) (**Figure 4E**). To evaluate the effectiveness of PD0325901 treatment to sustain MEK1/2-ERK1/2 pathway inactivation, the level of p(T202/Y204)ERK1/2 and total ERK1/2 from lung homogenates were quantified by western blot (**Supplementary Figure 3A**), revealing that *S. aureus* infected animals treated with PD0325901 had a significant reduction in the activation of ERK1/2 compared to both mock-infected and *S. aureus* infected vehicle treated groups (**Supplementary Figure 3B**). To determine if PD0325901 treatment and the reduction in neutrophils in the lung impaired bacterial clearance, CFU were measured in lung, liver, and spleen homogenates on day 1 after infection. Importantly, no significant difference in bacterial burden was observed the lung in infected mice treated with either vehicle or PD0325901 (**Figure 4F**). Further, CFU were below the limit of detection in liver homogenates from both vehicle (n=10) and PD0325901 (n=9) treated groups (data not shown), while only 1 of 9 spleen homogenates from the PD0325901 group had minimal CFU detected (8.888 x 10^3^ CFU/g, data not shown), and 0 of 10 from the vehicle-treated group. Together, these data demonstrate that treatment with the MEK1/2 inhibitor PD0325901 reduces neutrophil inflammation during acute infection in wild-type mice without impairing bacterial clearance during acute infection.

Although CF mouse models do not recapitulate many aspects of spontaneous lung disease found in human CF, CF murine models have been demonstrated to have increased susceptibility to pulmonary infections, including *S. aureus* (Guilbault et al., 2007; Li et al., 2017). CF mouse models also demonstrate innate immune cells that mount overly exuberant inflammatory responses to bacterial products, similar to immune cells isolated from PwCF (Bruscia et al., 2009; Oz et al., 2022). As we are proposing MEK1/2 inhibitors to be a potential therapy for PwCF, we sought to determine if a MEK1/2 inhibitor compound can dampen inflammation in the CF mouse model without permitting excessive bacterial growth. Therefore, we utilized the CF mouse model to further evaluate the effects of PD0325901 treatment during *S. aureus* infection. Similar to findings with wild-type mice, CF mice receiving PD0325901 treatment experienced significantly less body mass loss compared to vehicle-treated animals at day 1 after infection (**Figure 4G**). Bacterial burdens quantified in lung, liver, and spleen homogenates at day 1 in CF mice revealed there were no significant differences in bacterial clearance in the PD0325901 treated group compared to vehicle-treated controls (**Figure 4H**). Immunohistochemistry staining of CD45 to identify leukocytes in lung of infected CF mice at day 1 (**Figures 4I-L**) revealed a trend towards decreased inflammatory cell recruitment (**Figure 4M**) and neutrophil elastase (NE) in broncho-alveolar lavage fluid (BALF) (**Figure 4N**) in PD0325901-treated mice compared to vehicle, though not statistically significant. Levels of TNF and CXCL1 in BALF were below the limit of detection for both groups. Considered together, these data demonstrate that MEK1/2 inhibitors have beneficial anti-inflammatory effects *in vivo* that do not impair bacterial clearance in an experimental model of acute MRSA pulmonary infection.

## Discussion

Even though HEMT has significantly improved clinical outcomes and health in many PwCF, there is still a critical need for novel therapies for PwCF with established lung disease associated with chronic bacterial infection and inflammation. Access to HEMT therapy remains restricted based on eligibility of only specific *CFTR* mutations, insurance, and socioeconomic status, and HEMT is still not available to PwCF in many countries. In addition, chronic infections remain challenges in CF, even for PwCF on HEMT. According to patient registry data, in the USA the prevalence of *S. aureus* infections in PwCF has been over 60% since 2006 (Cystic Fibrosis Foundation Patient Registry, 2022). Due to early colonization and high prevalence of respiratory infections, acquired and intrinsic antimicrobial resistance of airway bacteria are straining our arsenal of therapeutic options. Novel therapeutic strategies to reduce detrimental inflammatory responses while combatting infection have major potential to slow pathology in CF, reducing symptom burden and enhancing patient longevity.

Our previous studies using non-CF human macrophages and wild-type mice demonstrated that MEK1/2 inhibitors modulated macrophage polarization *in vitro* and *in vivo*, and mitigated illness in wild-type mice when therapeutically delivered after initiation of experimental LPS-induced acute lung injury or *P. aeruginosa* pneumonia (Long et al., 2017a; Long et al., 2017b). Other non-CF models of infection and inflammation demonstrated complementary findings to our results, supporting the anti-inflammatory therapeutic potential of MEK1/2 inhibitors (Schuh and Pahl, 2009; Shi-Lin et al., 2015; Saint et al., 2016; Smith et al., 2016; Yu et al., 2022). However, the application of a MEK1/2 inhibitor in preclinical models of pulmonary MRSA infection, a major chronic pathogen in PwCF, has not been reported. Furthermore, there have been no studies investigating the effects of MEK1/2 inhibitors on CF immune cells, which mount aberrant anti-microbial and inflammatory responses (Bruscia et al., 2009; Oz et al., 2022), and thus may respond differently to immune modulatory therapy as compared to cells from healthy donors.

This study is the first to investigate the therapeutic potential and immunomodulatory roles of MEK1/2 inhibitors using human CF macrophages and neutrophils, and in an experimental murine MRSA infection model in wild-type and CF mice. The results from this study demonstrate that inhibition of the MEK1/2 pathway decreases LPS-induced production of IL-1β and secretion of IL-8 by CF macrophages, two key inflammatory mediators that potentiate TLR4-mediated recruitment of neutrophils and tissue damage. The results further demonstrate that MEK1/2 pathway inhibition also reduced TLR2/1- and TLR2/6-mediated production of IL-1β, suggesting that this pathway may be targeted to reduce inflammation due to either gram-negative or gram-positive infections. While reduction of pro-inflammatory mediators may limit inflammation associated tissue damage, there was also a moderate reduction in IL-10, which has anti-inflammatory effects. It was previously demonstrated that murine macrophages treated with the MEK1/2 inhibitor U0126 resulted in a decrease in *Il10* expression following Pam3CSK4 stimulation with a corresponding increase in *Il12p40 (Il12b)* expression (Groft et al., 2020). We did not observe significant reduction of *Il12a* or *Il12b* expression in macrophages treated with MEK1/2 inhibitors following either LPS, Pam3CSK4, or FSL1 stimuli, with modest trends towards increased expression. In the context of *S. aureus* infection IL-10, has been demonstrated to play a protective role in systemic acute infection but a detrimental effect during subcutaneous infection (Leech et al., 2017). Further, *S. aureus* metabolite reprogramming of macrophages to increase IL-10 has been identified as a contributing factor to facilitate persistent biofilm infection on prosthetic joints (Heim et al., 2020). On the other hand, IL-12 has been demonstrated to play a critical role in providing protective host defense during MRSA pneumonia (Nguyen et al., 2015). Given these results, we suspect while decreased IL-10 due to MEK1/2 inhibitor treatment may reduce some of the potential systemic anti-inflammatory effects of IL-10, this reduction may favor macrophage phenotypes less permissive to the establishment of chronic *S. aureus* infections. In addition, we suspect that IL-12-mediated host defense is not altered with MEK1/2 inhibitor therapeutic treatment. Future studies should further evaluate whether MEK1/2 inhibitor treatment alters the balance of IL-10 and IL-12-mediated inflammation and *S. aureus* host defense during pulmonary infection, including NK cell-mediated secretion of IFN, and macrophage responses to IFN stimulation (Nguyen et al., 2015). In addition, the data presented here indicate that inhibition of the MEK1/2 pathway does not decrease the phagocytic capacity of CF macrophages and neutrophils or affect phagosome acidification. This is consistent with a previous study utilizing the murine macrophage cell line RAW264.7 or human neutrophils reporting that addition of the MEK1/2 inhibitor PD0325901 increased *S. aureus* killing within the first hour of exposure by these phagocytes (Kurian et al., 2019). Finally, administration of a MEK1/2 inhibitor compound to mice at the time of MRSA pulmonary infection did not impair bacterial clearance or result in increased dissemination of infection at early time points. Instead, groups of mice treated with the MEK1/2 inhibitor had reduced body mass loss as a marker of illness with decreased neutrophil mediated inflammation compared to vehicle treated groups, and PD0325901 treated mice did not exhibit a loss of body mass or illness over a six day monitoring period, suggesting there was no resurgent infection. These new findings are consistent with previous reports demonstrating that MEK1/2-ERK1/2 pathway inhibition reduces airway epithelial cell, including CF epithelial cell, production of IL-8 (Bhattacharyya et al., 2011; Cormet-Boyaka et al., 2012), and that inhibition of the MEK1/2-ERK1/2 pathway can prevent airway epithelial cell CFTR degradation (Xu et al., 2015; Wellmerling et al., 2022). Combined, these new data support the hypothesis that MEK1/2 inhibitor compounds exert anti-inflammatory effects without impairing host defense mechanisms, and thus may have a high potential as therapies for PwCF.

There are several limitations of the current study. First, we did not perform comparative studies in human macrophages or in murine infection models to evaluate whether activation of the MEK1/2-ERK1/2 pathway was significantly different between non-CF and CF samples; the goal of our study was to evaluate the translational potential of MEK1/2 inhibitor compounds in the context of CF, and not a comparative examination of potential non-CF to CF differences. However, our data demonstrate that both TLR2 and TLR4-dependent stimuli activate the MEK1/2-ERK1/2 pathway in CF macrophages, and that activation can be reduced with MEK1/2 inhibitor compounds. In studies with human CF cells, the majority were obtained from donors with at least one copy of F508del, which is the most commonly occurring *CFTR* mutation. Future studies should critically evaluate whether MEK1/2 inhibitors have differing effects on human CF immune cells obtained from individuals harboring different classes of *CFTR* mutations. This information would be extremely important to understand whether PwCF not eligible for CFTR modulator therapies would benefit from a potential MEK1/2 inhibitor therapy. Second, while the CF mouse model utilized recapitulates many phenotypes of CF, the mouse does not reproduce all aspects of human CF pulmonary disease, such as spontaneous airway disease and airway acidification defects. In addition, the MRSA infection employed results in a modest and transient disease, which does not fully capture the chronic infections commonly found in CF, especially those in which *S. aureus* infections may be maintained by biofilms (Kolpen et al., 2022). We also note the limitation that CF mice were only evaluated at one time point, and that it is possible that bacterial burden could rebound later in the inhibitor treated mice. In addition, evaluation of whether bacterial burden, or the persistence of *S. aureus* infection in the nasal cavity or upper respiratory tract was altered by MEK1/2 inhibitor treatment would provide additional information on the potential safety of this treatment. Given these limitations., this murine model is still an important tool for the development of preclinical studies as it models complex immune cell interactions with bacteria in the lung. Future studies utilizing this murine model should investigate infection with clinical CF *S. aureus* isolates, which have recently been reported to withstand killing by higher concentrations of hypochlorous acid and have a potential *in vivo* fitness advantage compared to non-CF strains, and therefore may have different responses to therapeutic treatment with a MEK1/2 inhibitor compound (Jennings et al., 2023). While our approach utilized the MRSA USA300 strain, which is associated with community-acquired human disease, CF clinical isolates of *S. aureus* may have significant genetic and phenotypic differences to produce different inflammatory host responses. For example, infection with *S. aureus* small colony variants (SCV) have been demonstrated to elicit worse outcomes in PwCF and infection induces an increased inflammatory response compared to non-SCV *S. aureus* infection in the CF rat (Wolter et al., 2013; Bollar et al., 2022). Overall, the current findings support a rationale for future investigative preclinical studies utilizing CF models, such as the CF rat (Bollar et al., 2022; Henderson et al., 2022), that more faithfully recapitulate CF pulmonary disease in conjunction with chronic infection or poly-microbial infection.

The translational potential of this work is highly relevant, as a recent human clinical trial (NCT04776044) has tested the therapeutic potential of the MEK1/2 inhibitor compound ATR-002 in the context of SARS-CoV-2 infection based in part on *in vitro* preclinical experimental studies (Schreiber et al., 2022b). Future human clinical trials with ATR-002 may provide additional data to help determine the safety and efficacy of MEK1/2 inhibitor compounds during human respiratory infections and could serve as a foundation for evaluating the safety for translational application in PwCF. Significantly for CF, there is evidence that the ATR-002 compound has direct antibacterial effects on gram-positive organisms, including *S. aureus* (Bruchhagen et al., 2018). Future preclinical studies for CF should be designed to harness the combined antimicrobial and potential anti-inflammatory effects of this MEK1/2 inhibitor compound. In summary, this study provides the first data evaluating the immunomodulatory and therapeutic potential of MEK1/2 inhibitor compounds on CF immune cells and in the CF airway. The findings support the hypothesis that inhibition of the MEK1/2 pathway is a therapeutic target to reduce inflammation without impairing cellular and organism level host defense mechanisms. Future preclinical studies to provide additional rigorous assessment of the impact on inflammation, host defense, and the antimicrobial potential of MEK1/2 inhibitor compounds should be performed with the goal of developing a path to human CF translational studies.

## Data availability statement

The original contributions presented in the study are included in the article/**Supplementary Material**. Further inquiries can be directed to the corresponding author.

## Ethics statement

The studies involving humans were approved by The OSU Institutional Review Board. The studies were conducted in accordance with the local legislation and institutional requirements. The human samples used in this study were acquired from de-identified biospecimens collected by the Cure CF Columbus Research Development Program Translational Core. Written informed consent for participation was not required from the participants or the participants’ legal guardians/next of kin in accordance with the national legislation and institutional requirements. The animal study was approved by The OSU Institutional Animal Care and Use Committee. The study was conducted in accordance with the local legislation and institutional requirements.

## Author contributions

MD: Data curation, Writing – review & editing. GS: Writing – review & editing, Conceptualization, Investigation, Methodology, Supervision. EZ: Methodology, Writing – review & editing, Conceptualization, Investigation, Supervision. KH: Funding acquisition, Writing – review & editing. WL: Methodology, Writing – review & editing. AM: Funding acquisition, Resources, Writing – review & editing. EH: Data curation, Funding acquisition, Methodology, Resources, Writing – review & editing. ML: Conceptualization, Data curation, Funding acquisition, Investigation, Methodology, Project administration, Resources, Writing – original draft, Writing – review & editing.
