# Supplementary figures and images for: MEK1/2 inhibition decreases pro-inflammatory responses in macrophages from people with cystic fibrosis and mitigates severity of illness in experimental murine methicillin-resistant Staphylococcus aureus infection
Source: Front Cell Infect Microbiol. 2024 Jan 30;14:1275940. doi: 10.3389/fcimb.2024.1275940 (PMC10861668; doi:10.3389/fcimb.2024.1275940)

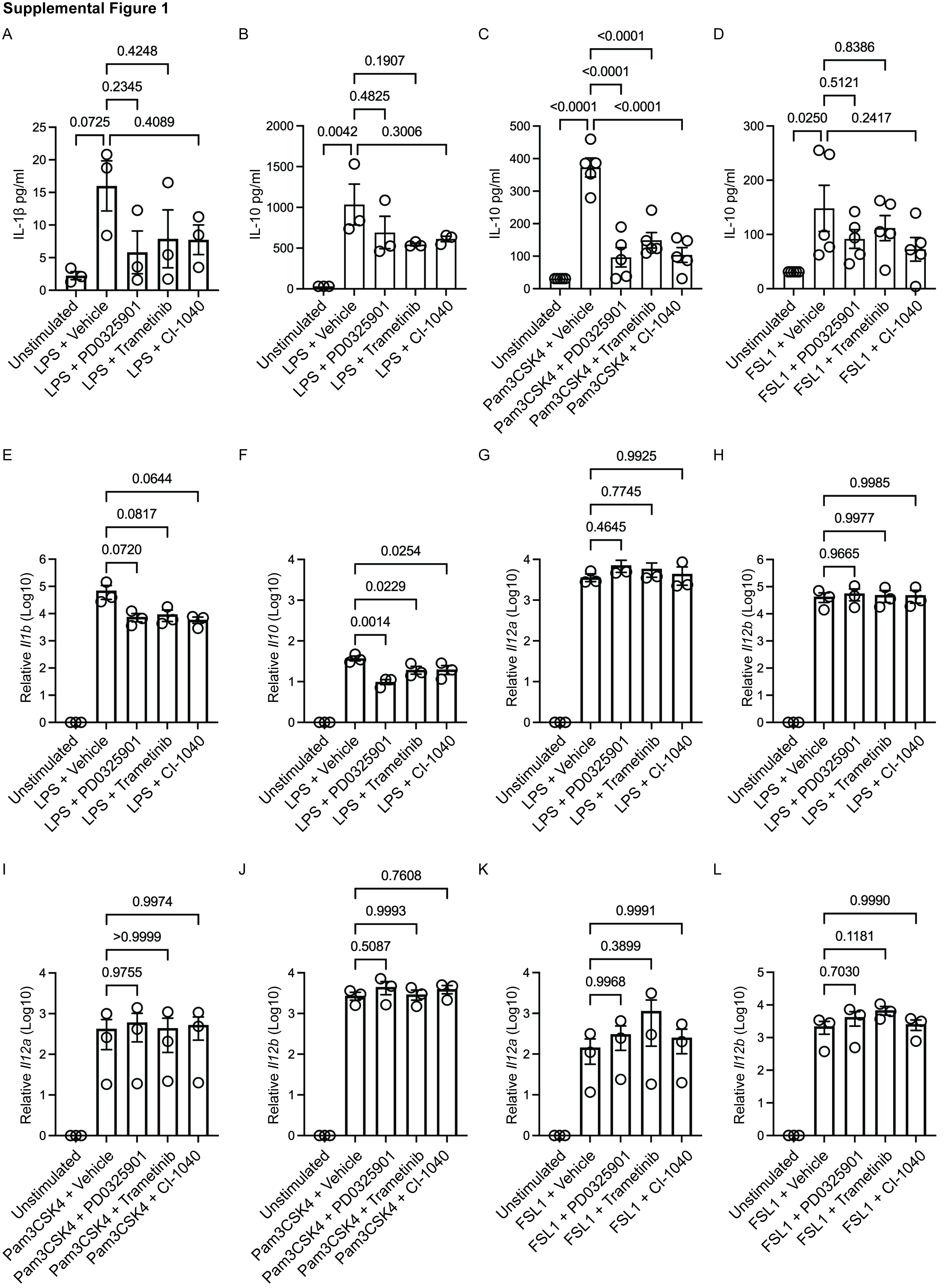

Supplement: Supplementary file 1 [file Image_1.tif]

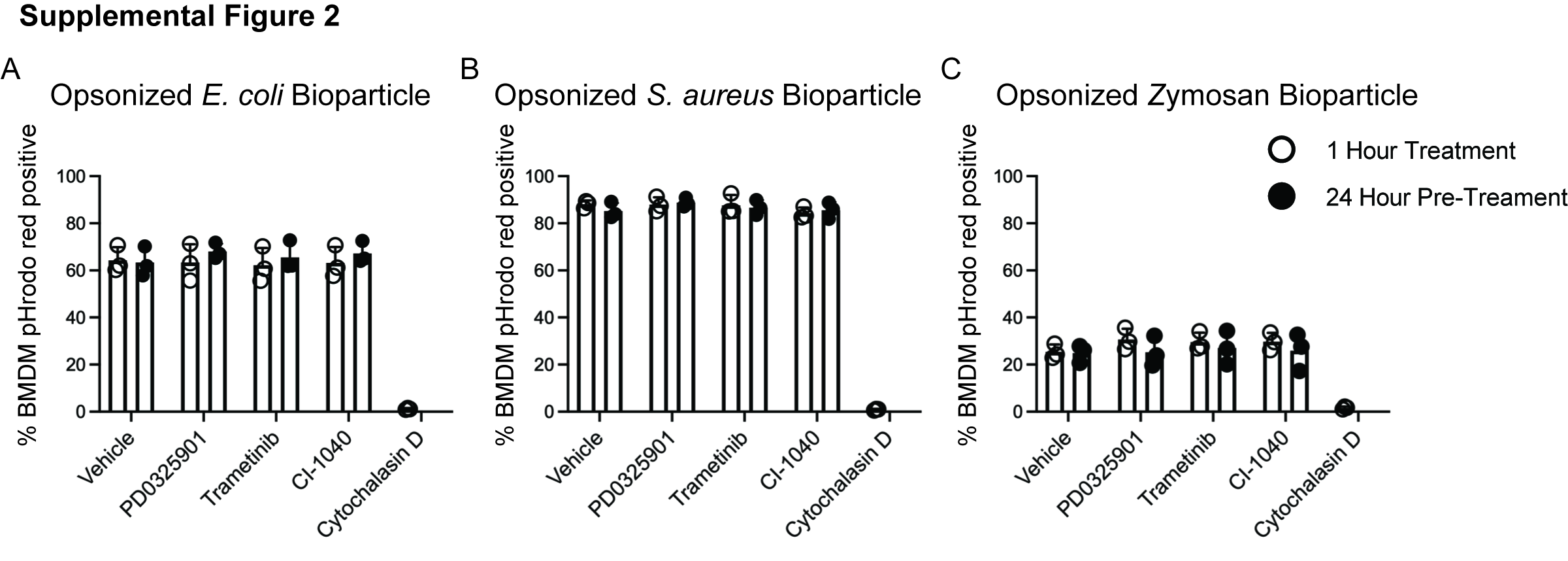

Supplement: Supplementary file 2 [file Image_2.tif]

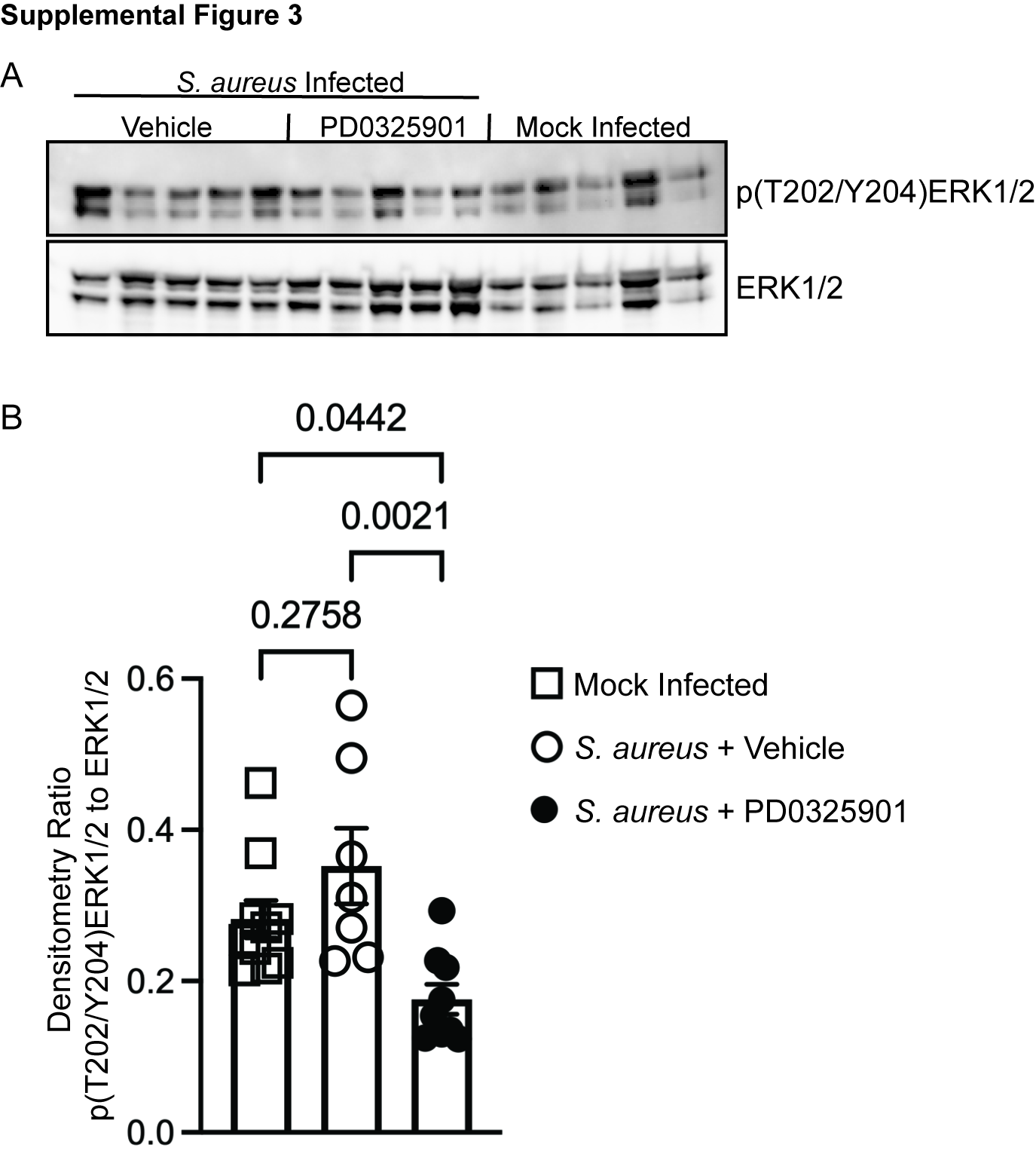

Supplement: Supplementary file 3 [file Image_3.tif]
