# Supplementary material for: MEK1/2 inhibition decreases pro-inflammatory responses in macrophages from people with cystic fibrosis and mitigates severity of illness in experimental murine methicillin-resistant Staphylococcus aureus infection
Source: Front Cell Infect Microbiol. 2024 Jan 30;14:1275940. doi: 10.3389/fcimb.2024.1275940 (PMC10861668; doi:10.3389/fcimb.2024.1275940)
Supplement: Supplementary file 4 [file Table_1.docx]

**Supplemental Figure 1**. **Effects of MEK1/2 inhibitors on macrophage cytokine production.** The levels of cytokines secreted into the supernatant from human CF macrophages stimulated for 4 hours with either (A-B) LPS, (C) Pam3CSK4, or (D) FSL1 was quantified by ELISA for (A) IL-1beta and (B-D) IL-10. Murine bone marrow derived macrophages (E-L) from wild-type mice were stimulated for 4 hours with either (E-H) LPS, (I-J) Pam3CSK4, or (K-L) FSL1 with the addition of vehicle or MEK1/2 inhibitor compounds. Gene expression by qPCR was used to measure relative compared to untreated samples. Data are the mean $\pm$ SEM from n=3-5 biological replicates. Statistical analyses performed with One-way ANOVA and Tukey’s multiple comparisons; (E-L) samples were compared to stimulus + vehicle condition.

**Supplemental Figure 2. Prolonged treatment of macrophages with MEK1/2 inhibitors does not impair phagocytosis.** Murine bone marrow derived macrophages from wild-type mice were pre-treated with MEK1/2 inhibitors or a vehicle control for 24 hours before initiation of phagocytosis assays using opsonized pHrodo red bioparticles of (A) *E. coli*, (B) *S. aureus*, or (C) zymosan. Phagocytosis was performed in media containing fresh vehicle or MEK1/2 inhibitor compounds. In contrast to the 24 hour pre-treatment conditions, additional macrophages were only exposed to a MEK1/2 inhibitor or vehicle compound during the 1 hour phagocytosis incubation period. Macrophages exposed to cytochalasin D only during the 1 hour phagocytosis incubation period were used as a control to inhibit phagocytosis. Data are the mean and standard deviation from n=3 biological replicates.

**Supplemental Figure 3. Treatment with PD0325901 reduces ERK1/2 activation in the lung following *S. aureus* infection.** Wild-type C57BL6/J mice were mock-infected or infected with *S. aureus* and provided i.p. treatment with either vehicle or PD0325901. Protein lysates from lung homogenates were collected on day 1 following infection. (A) Representative western blot from one of two independent experiments demonstrate levels of p(T202/Y204)ERK1/2 and total ERK1/2; each lane is lung homogenate from an individual mouse. (B) Densitometry quantification using ImageJ includes samples from two independent experiments: n=10 mock-infected, n=7 *S. aureus +* Vehicle (two samples excluded because protein lysates had significant degradation, one sample excluded as its densitometry elevated ratio of 0.946 was identified as a statistical outlier using GraphPad Outlier calculator’s Grubbs’ test of Alpha = 0.05), n=9 *S. aureus* + PD0325901. Statistical analysis used One-Way ANOVA with Tukey’s multiple comparisons.
