# Supplementary material for: MEK1/2 inhibition decreases pro-inflammatory responses in macrophages from people with cystic fibrosis and mitigates severity of illness in experimental murine methicillin-resistant Staphylococcus aureus infection
Source: Front Cell Infect Microbiol. 2024 Jan 30;14:1275940. doi: 10.3389/fcimb.2024.1275940 (PMC10861668; doi:10.3389/fcimb.2024.1275940)
Supplement: Supplementary file 5 [file Table_2.docx]

Supplemental Table 1: Demographics of Blood Donors.

| **Sex** | **CFTR Genetics** |
| --- | --- |
| Female | F508del/F508del |
| Female | F508del/R751L |
| Male | F508del/2184delA |
| Male | F508del/F508del |
| Female | F508del/F508del |
| Male | F508del/F508del |
| Male | R117H/W1282X |
| Female | F508del/F508del |
| Female | F508del/G551D |
| Female | F508del/c.2848_2849ins~1.6kb |
| Male | F508del/F508del |
| Female | F508del/F508del |
| Female | F508del/G551D |
| Female | F508del/F508del |
| Female | F508del/3600G>A |
| Female | F508del/R117H |
| Female | F508del/G551D |
| Male | F508del/F508del |

Supplemental Table 2: List of antibodies used for western blot.

| **Target** | **Company** | **Species** | **Catalog #** |
| --- | --- | --- | --- |
| GAPDH (14C10) | Cell Signaling | Rabbit | 2118 |
| P44/42 MAPK (ERK1/2) (137F5) | Cell Signaling | Rabbit | 4695 |
| p-P44/42 MAPK (ERK1/2) (T202/Y204) | Cell Signaling | Rabbit | 9101 |
| IL-1β (D3U3E) | Cell Signaling | Rabbit | 12703 |
| CD45 | abcam | Rabbit | ab10558 |

Supplemental Table 3: List of ELISA Kits.

| **Target** | **Company** | **Catalog #** |
| --- | --- | --- |
| Human CXCL8/IL8 | Biotechne/R&D Systems | DY208-05 |
| Human IL-1beta/IL-1F2 | Biotechne/R&D Systems | DY201-05 |
| Human IL-10 | Biotechne/R&D Systems | DY217B-05 |
| Human IL-12 p70 | Biotechne/R&D Systems | DY1270-05 |
| Human TNF-alpha | Biotechne/R&D Systems | DY210-05 |
| Mouse Neutrophil Elastase/ELA2 | Biotechne/R&D Systems | DY4517-05 |

Supplemental Table 4: List of primer probes for qPCR.

| **Target** | **Company** | **Catalog #** |
| --- | --- | --- |
| *Hprt* | Life Technologies | Mm03024075_m1 |
| *Il1b* | Life Technologies | Mm00434228_m1 |
| *Il10* | Life Technologies | Mm01288386_m1 |
| *Il12a* | Life Technologies | Mm00434169_m1 |
| *Il12b* | Life Technologies | Mm01288989_m1 |
